# Home Delivery Medicament Program: access, inactivity and cardiovascular risk**^1^**

**DOI:** 10.1590/1518-8345.1038.2810

**Published:** 2016-10-10

**Authors:** Roque da Silva Araújo, Edna Apparecida Moura Arcuri, Victor Cauê Lopes

**Affiliations:** 2 MSc, RN, Unidade Básica de Saúde Anhanguera, Prefeitura Municipal de São Paulo, São Paulo, SP, Brazil.; 3 PhD, Professor, Centro de Pós-Graduação e Pesquisa, Universidade Guarulhos, Guarulhos, SP, Brazil.; 4 MSc, Professor Assistente, Faculdades do Vale do Juruena, Juína, MT, Brazil.

**Keywords:** Patient Compliance, Government Programs, Program Evaluation, Public Health, Health Promotion

## Abstract

**Objective:**

to verify causes of inactivity in the Home Delivery Medicament Program, as referred by users from a Primary Health Care Service in São Paulo, comparing them to the causes registered in the program and analyzing them in the theoretical model Concept of Access to Health.

**Methods:**

cross-sectional study, interviewing 111 inactive users; and documentary study in the program records.

**Results:**

half of the users did not know the condition of inactivity. Discrepancies were found between the user's and the program's information, observing different levels of agreement: Absence of physician and administrative staff member 0%; Transfer to other service 25%; Death 50%; Option to quit 50%; Address change 57% and Change in therapeutic schedule 80%. The users' feeling of accepting the program was observed. In the health access concept, inactivity can be explained in the information dimension, in the degree of asymmetry between the patient's and the health professional's knowledge, identified through the indicators: education, knowledge and information sources.

**Conclusions:**

due to the low education level, the user does not assimilate the information on the steps of the program flowchart, does not return for the assessment that guarantees its continuity. Consequently, (s)he stops receiving the medication and spends a long time without treatment, increasing the cardiovascular risk of hypertensive (92% of the sample), diabetic (44%) and dyslipidemic patients (31%).

## Introduction

The change in the morbidity and mortality profile due to chronic illnesses and cardiovascular complications, such as acute myocardial infarction and stroke, has redirected public and scientific health care policies^1^. Concomitantly with the international guidelines^2^ ^-^ ^3^, with the attempts to find evidence in order to understand the low treatment compliance rates^4^ and with governmental programs and the supply of services to reduce the high prevalence rates of these problems, several authors have made efforts to understand and discuss the Health Access concept.

In the 1960's, challenges emerged in the field of public or private health policies, with heated discourse by economists about the uncertainties in the economic wellbeing of medical care. The literature in the 1970's was enriched by reflections on the concept of health access, in which Donabedian's work became a landmark for experts on the theme^5^. The focus turned to the characteristics of the population, emphasizing the importance of individual determinants like income, health coverage, attitudes towards care and social structure^6^.

The concept of health access gained consistency through the aggregation of socio-organizational attributes like the social, cultural and educational condition, variables that could be assessed by means of outcome indicators of the user's passage through the system, such as user satisfaction, an attribute that was also highlighted at the start of the 1980's^7^.

Different authors have revised the concept of health access in the 21^st^ century^8^ ^-^ ^10^, considering four dimensions that can be assessed by means of process and outcome indicators, which help to judge the conditions of equity or inequality in the access to health: availability; acceptability; capacity and information^11^.

Recent studies on medication access are scarcer, although the World Health Organization (WHO) has published on the theme^12^ ^-^ ^13^.

The objective in this study is to understand why the Home Delivery Medicament Program, an example of an easily available access to medication treatment, registers high inactivity rates. The authors considered that analyzing the reasons for the apparent dropout of the program, in the light of the four dimensions of the health access concept, could result in a comprehensive view of the variables involved.

Considering that Arterial Hypertension is the main cardiovascular risk factor, particularly affecting the elderly population all over the world, and in combination with Diabetes Mellitus and other factors aggravates the risk of cardiovascular complications^14^, in 2005, the São Paulo Municipal Health Department implemented the Home Delivery Medicament Program (HDMP). The objective was to guarantee medication access and continuing care for patients suffering from these conditions, through the home delivery of sufficient drugs for 90 days^15^.

At first, the HDMP prioritized Diabetes and/or Arterial Hypertension patients, in stable and clinically controlled conditions, monitored at Primary Health Care Units (PHCU). Later, patients with dyslipidemias and thyroid problems were included.

In a PhD study on medication access by the low-income population in a neighborhood of São Paulo, it was concluded that picking up medication at the desk of the PHCU demanded time, money, frustration and increased the rates of absence from work. Besides receiving the drug at home, the users were guaranteed a scheduled return appointment and further tests for control. The author concluded that the HDMP resulted in lesser risk of problems, a better bond with the team, in addition to the fact that the users felt more valued and taken care of (Unpublished data). Nevertheless, in recent years, the leaders have been facing high levels of inactivity in the program, with information on why the users dropped out of the program.

The objective in this study was to identify the users' reasons to drop out of the Home Delivery Medicament Program, to compare them to those registered electronically at the PHCU in the program files and to analyze the results in view of the current Health Access concept.

## Method

Cross-sectional, analytic, documentary field study, undertaken at a Primary Health Care Unit in the North of the City of São Paulo.

*Flowchart of Home Delivery Medicament Program:* the authors found it essential to start the methodological trajectory by analyzing the flowchart of the program, distinguishing the following steps: a) during a clinical assessment as part of a routine appointment, the consulting physician includes patients who comply with the program requisites. b) after the assessment, with the standardized prescription of the program at hand, the patient goes to the pharmacy, where the technician includes him/her in the GSS/Medicament at Home system with the patient's consent. c) the user receives the program orientations and prescribed drugs to be taken within the first 15 days, the deadline for the first set of drugs sufficient for 90 days to reach his home by mail. A second set is sent for yet another 90 days, totaling 180 days or six months. Before the end of this period, however, the user should again consult the physician, thus guaranteeing the non-interruption of the medication. (S)he should return within 75-90 days for a group or individual assessment with the Nurse and/or Pharmacist to maintain an active status in the Medicament at Home Program. This assessment should be registered in his/her patient history. During the consultation after six months, the physician decides on whether the user should continue in the Program, with his/her agreement, or formulates the clinical reason for the lack of maintenance. If the patient does not return, (s)he becomes inactive^6^.

*Sample:* Initially, all 136 users registered as inactive in the program were included; nevertheless, four had died, three had moved to other regions, one was active in the program (registration error) and 24 inactive users could not be located, resulting in a sample of 104 interviewed participants and rates of 111 compared indices (04 deaths and 03 address changes). *Ethical evaluation:* Initially, the project received approval from the Ethics Committee of the São Paulo Municipal Health Department, opinion 59/11, for face-to-face interviews. In view of difficulties, the committee authorized telephone interviews (opinion 272/11). 

*Participant recruitment:* the telephone number registered in the user's enrollment was used. When the telephone no longer corresponded to the number registered at the PHCU and the person who answered was unknown, the researchers investigated the registration on the SUS (public health system) card or the local telephone records, looking for the subscriber's name or the address. If that did not result in contact, the address was visited. If that was not possible, the user was excluded from the research.

*Data collection:* in the first phase, the information in the program files about the reasons for inactivity (documentary study) was collected and registered. In the second phase, the interviews were held to verify the reasons for dropping out the users had indicated. The semistructured interview technique was applied to the user or his/her caregiver, in case of physical or intellectual disability, addressing topics related to the sociodemographic variables, which were fundamental to use the analysis model adopted, the Health Access concept. The participant's record at the PHCU was used as a data source, further completing the data during the interview. 

The interview was mainly focused on the user's reasons to drop out of the program. All possibilities were explored for the participant to feel at ease to explain the reasons for his/her inactivity and to identify his/her feelings about the program. It is highlighted that, although the interviewer was the manager of the PHCU in question, difficulties to establish telephone contact were expected, which are common in recent times, for reasons of social protection as well as information constraints. Nevertheless, mentioning some data from the patient history, demonstrating knowledge about the user was a strategy that facilitated the initial contact.

*Data analysis:* to understand and discuss the reasons for activity, it was fundamental to analyze the users' inclusion in the HDMP flowchart, the sociodemographic characteristics and to analyze the results based on the concept of Health Access, described in four dimensions^4^. These were presented here because they represented an interesting theoretical model to discuss the data after the most recent review by Brazilian authors^11^.

*Availability:* geographical relation between the services and the individual, such as distance and transportation options; relation among type, range, quality and quantity of health services delivered. Indicators: type of service used (hospital, medical, dental, emergency, home care), place where the care was provided (home, consultation room, clinic, hospital), purpose of care (preventive, curative), % of the population at risk who visited a physician or not within a given interval, number of beds, equipment.

*Payment power:* relation between cost of health service use and individuals' payment capacity. Indicators: income, sources of income, health insurance coverage, characteristics of regular care source, direct and indirect costs on health.

*Information:* degree of asymmetry between patient and health professional's knowledge. Indicators: education, knowledge and information sources.

*Acceptability:* nature of services provided and perception of the services by individuals and communities, influenced by cultural and educational aspects. Indicators: beliefs and attitudes towards health, knowledge and information sources about health care, anxiety related to health, trust in the health system.

The data were stored and analyzed using descriptive statistics with the help of SPSS, version 17.0, which facilitate the analysis of the research variables. 

## Results

During the interviews, data were revealed that responded to the objectives. The users' participation was heated by the fact that many of them did not know about their exclusion from the Home Delivery Medicament Program and the need to express positive feelings about it. Table 1 displays the subjects' sociodemographic data.

**Table 1:** User frequency in function of age range, sex and education. São Paulo, SP, Brazil, 2011.

		N	%
Age Range			
	< 50	13	11.7
	50 to 59	23	20.7
	60 to 69	28	25.2
	70 to 79	32	28.8
	≥ 80	15	13.5
Sex			
	Male	35	31.5
	Female	76	68.5
Education			
	Never been to school	24	21.6
	Primary	78	70.3
	Secondary	8	7.2
	Higher	1	0.9

As verified in Table 1, women are predominant among the users. The population sample includes mainly elderly people, with two-thirds of the participants being over 60 years of age and having a low education level. 

**Figure 1:**
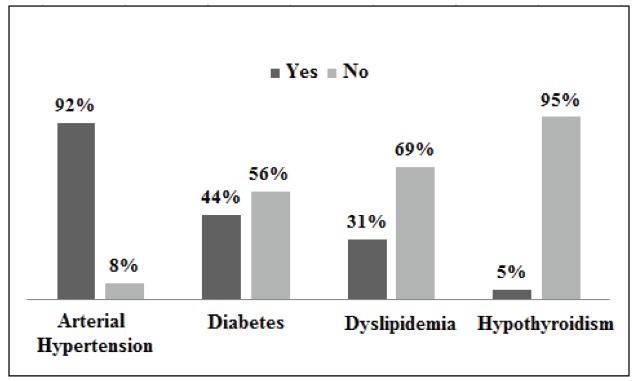
Prevalence of diseases prioritized in the program among users registered in the Home Delivery Medicament Program. São Paulo, SP, Brazil, 2011

Figure 1 displays the diagnoses that led to the users' inclusion in the program, making it easier to observe possible associations of cardiovascular risk factors (Figure 1). It reveals that the inactive users associated several cardiovascular risk factors, particularly hypertension and diabetes, besides dyslipidemias. The users spend long periods without medication, due to their exclusion from the program, according to data in Table 2.

Yes = presence of the disease; No = absence of the disease.

**Table 2:** Period of previous consultation. São Paulo, SP, Brazil, 2011

Time of previous consultation	N	%
1 to 3 months	66	63.5
4 to 6 months	20	19.2
7 to 9 months	16	15.4
10 to 12 months	01	1.0
More than 12 months	01	1.0
Total	104	100

The data in Table 2 indicate a gap in the medication treatment, considering that the delivery of the drugs was interrupted for all inactive users after 90 days. They expressed the reasons that made it impossible to schedule the consultations, such as difficulty to leave their home, lack of appointments for 34.2% of the inactive users, and long queues. The participants' feelings about the program were expressed strongly (Table 3).

**Table 3:** Users' feelings about the Home Delivery Medicament Program. São Paulo, SP, Brazil, 2011

Feelings	N	%
I liked it, because it is difficulty for me to leave my home.	29	27.9
I liked it, because it is easy to receive the medication at home.	56	53.8
I liked it, because of the certainty that I will not fall short of the medication.	15	14.4
I did not like it because not all the drugs I need are delivered.	04	3.8
Total	104	100.00

It is noteworthy in Table 3 that 96.2% of the users expressed positive feelings about the HDMP, half of them due to the ease to receive the drugs at home. The reasons for inactivity at the PHCU were registered in eight categories, which were compared with the reasons the users had indicated. Table 4 displays the absolute frequency of the two variables compared for each category and the relative frequency of the level of agreement between them.

**Table 4:** Agreement between Users and Management Program records about reasons for inactivity. São Paulo, SP, Brazil, 2011

Reasons	Users	PHCU	N	% agreement
Change of address of user	4	7	7	57%
Transfer of user to other unit	1	4	4	25%
Change of therapeutic scheme	4	5	5	80%
Absence of physician to renew prescription	0	14	14	0%
Absence of administrative staff member	0	7	7	0%
Absence of information about user	24	50	50	48%
Death	2	4	4	50%
Option of user to no longer participate	11	20	20	55%
Total			111	

The lack of agreement between the HDMP records and reports of the inactive users in Table 4 evidence gaps to be identified in the program evaluation. In view of the relevant discrepancy between the information compared, the users' actual reasons are described for the main categories:

The category "Not informed" was registered in the program registers at the PHCU for 46 inactive users. It was verified that: 01 was due to death: 03 to address change: 01 change in therapeutic scheme; 10 problem in medical HR; 01 problem in administrative HR; 24 to lack of information and 06 to the option no longer to participate in the HDMP.

The management report registered 04 deaths versus 02 actual deaths; "Address change" was registered for 22 users in the program records, while the users reported: 01 death; 04 address change; 01 transfer of PHCU; 02 absence of medical HR; 01 administrative problem; 12 lack of information and 01 to the user's option.

The reason "Transfer of user to another unit" was indicated for 02 inactive users in the management report, considering that 01 agreed and the other decided that he no longer wanted to participate in the HDMP. The "change of therapeutic scheme" was registered for 11 inactive participants in the management report, but the participants reported: 04 due to change of therapeutic scheme, 01 due to administrative problem, 04 due to lack of information from the user and 02 to the patient's choice no longer to participate in the HDMP. The latter category, not participating, was registered for 28 users in the management report, considering that the subjects informed: 02 due to transfer to another PHCU, 02 to absence of medical HR, 04 to administrative problem, 09 to lack of user information and 11 to the user's option not to participate in the HDMP. 

## Discussion

From the methodological viewpoint, one could expect that the telephone interview would restrict the information. That did not happen, probably because many participants were informed of their inactive status in the HDMP when they received the ethical details of the protocol, such as the aspect inherent in the consent to participate in the research. The words "drop-out" or "no longer attending" the program were used to clarify the condition of "inactive", due to the education level. The discovery of this condition provoked feelings that facilitate the information needed to reach the objectives. The feeling of restricted access made some users feel abandoned, making them express their feelings about the HDMP and the information needed. 

Concerning the sociodemographic variables, the gender data are in line with other studies undertaken at health services, where the number of female users surpasses that of male ones^16^, possibly due to the greater availability, considering that men tend to work until more advanced ages. Nevertheless, even after retiring (70 years), men do not reach the same compliance observed in women. Data from the Brazilian Institute of Geography and Statistics do not appoint differences between the sexes in the region studied^17^.

The advanced age of many inactive users was no surprise, due to the high prevalence of hypertension and diabetes mellitus after the age of 60 years, diseases prioritized in the program. Although the estimated prevalence of hypertension of 35.8% in men and 30% in women^14^, there are no exact studies that evidence a relevant increase in these percentages after the ages of 50-60 years.

The low education level found may have influenced the difficulty to understand the MHP orientations and standards, in line with other data found in São Paulo for hypertensive elderly, many of whom were illiterate^18^. The education level is a variable highlighted in reviews on treatment compliance and worsening of chronic illnesses, and directly related to the socioeconomic conditions of the populations^19^.

In the analysis of education results in the health access concept presented in the method^11^ the dimension *information*, the degree of asymmetry between the patient and the health professional's knowledge, is identified through the indicators: *education, knowledge* and *information sources*. A degree of asymmetry exists between the knowledge of the inactive users and the PHCU professionals. The low education level does not permit further understanding of the steps in the HDMP flowchart on the day of inclusion. The analysis reveals weakness in the educational aspects, absence of background explanations, of illustrative material to facilitate the understanding and alert the user to the data; besides suggesting the professionals' lack of engagement and competency to check the user's return of the information; and absence of the community health agent.

Concerning the dimension *availability*, in the category *geographical region,* although public transportation is available, when the region is large, with distant homes, this causes difficulties for the user and is the reason why the HDMP was created, in order to deliver drugs to the users' homes.

As for the indicator *type of service*, delivery by mail was successful, highlighting that its efficiency is known all over the State of São Paulo. In the indicator *percentage of risk population that visited a physician or not in a given interval*, the data reveal that, besides lack of understanding of the return date, a severe human resource problem exists to respond to the demand of unscheduled elderly and long waiting periods when scheduled beyond the HDMP.

In the dimension *"payment power: relation between cost of health service use and individuals' payment capacity"*, it is highlighted that the program was offered indistinctly to the subjects included for diagnostic reasons, although many PHCU users from the peripheral regions of the city belong to poorer population groups. 

Another dimension in the analytic framework is *acceptability,* the nature of the services provided and the individuals and communities' perception of the services; influenced by cultural and educational aspects. Indicators: *beliefs and attitudes concerning health, knowledge and information sources about health care, anxiety related to health, trust in health system.* The feelings the users expressed evidence positive attitudes towards HDMP and huge satisfaction to receive the drugs at home, even when not receiving the prescribed drugs, but beyond the program. Besides the comfort, they addressed the safety and trust in the receipt of this benefit.

The analysis of the study results suggests, from the perspective of the *health access concept*, that the main dimension to be considered in the users' inactivity is *Information*, in view of the existing *asymmetry* between the knowledge of the users and health professionals from the PHCU. In addition, the strongest indicators were *education, knowledge and information sources*, important determinants to understand the steps in the HDMP flowchart, essential to prevent inactivity.

The HDMP started one year after WHO had declared that: "access to essential medicines is missing for more than two billion people and the lack of these medicines causes avoidable suffering, such as disease, pain, fear, lack of dignity". These assertions involve authors who claim that "access to medicines, especially those considered lifesaving, are part of citizens' right to the highest possible level of health, with duties attributed to the State and responsibility by the pharmaceutical companies"^20^.

The analysis of the literature indicates a trend to medication distribution at home, resulting in advantages for treatment compliance and cost-benefit, comfort and safety for consumers. In a recent North American study, the compliance with patients with diabetes, hypertension and high cholesterol to the drug treatment was compared when distributed at the pharmacy or at home, considering approximately 150,000; 615,000 and 359,000 patients, respectively. Using multivariate logistic regression, excluding variables to assess the impact of the distribution channel on medication adherence, control of demographic differences, low income, consequences of diseases and drug use standard, the authors concluded that medication at home can influence compliance^21^.

When discussing the human right to medication access and in view of the undeniable vulnerability of not granting this right to thousands of people around the world, in 2013, Moon considered the need to clearly establish the responsibilities of the government and the pharmaceutical industry and criticizes the weakness of terms in guidelines in the area, which "have to" instead of "need to"^22^. Hence, medication access and dispensation are still themes that require attention^23^.

In the same year as WHO's 2008 declaration, Brazilian authors examined the continuing use of hypertension medicines in adults and elderly in the South and Northeast of Brazil: use in 87% of 4003 elderly, with stronger associations for non-interruption among participants with high education levels, better economic conditions and greater compliance with PHCU programs. Nevertheless, considering the two regions studies, the results reveal inequity in the health access, strengthening the need to improve it, mainly for the low-income population^23^.

One important limitation in this study is the lack of data on the morbidity and mortality at PHCU or the worsening of the users' clinical condition, especially of the inactive users in the HDMP, which impedes associations between the interruption of the drugs, increased cardiovascular risk and worsening of the clinical condition. Nevertheless, the analysis of the users' educational level calls for attention to the interruption of anti-hypertensive drugs in elderly patients who associate several risk factors for cerebrovascular accident and stroke, such as hypertension, diabetes, dyslipidemias, among other modifiable risk factors (unbalanced calorie intake, sedentariness, obesity, smoking). This concern is in line with authors who observed associations between lower education level and cardiovascular complications in patients with diagnoses similar to the present study^24^ ^-^ ^25^. Therefore, the hypothesis is raised that a substantial part of inactive users who interrupt the drugs is vulnerable to cardiovascular complications.

The findings of the confrontation between data from the users and HDMP records arouse reflections: the program can only be efficient if operated in a staged process, the most relevant stage being the training of all employees involved. The users' education needs to be permeated by the continuing assessment of their understanding about the steps in the HDMP flowchart, with a view to avoiding the interruption of the treatment. 

The relevant number of inactive users without justifiable reasons indicates a lack of organization and technical and administrative effort at the PHCU, besides imprecise data in the HDMP records. The analysis of the access concept in this study reveals positive steps in the program concerning management initiatives in the public policies, including guarantees that the drugs reach the users' home and high levels of user satisfaction. Inactivity is associated with the fact that the HDMP users' precarious educational conditions are ignored.

## Conclusion

This study identified discrepancies between the reasons for inactivity registered in the HDMP and the reasons the users indicated. The main cause of inactivity is related to the users' difficulty to understand the steps in the program flowchart, due to the precarious education level. Administrative gaps were observed at the PHCU in relation to the program flowchart. The data analysis in the light of the *Health Access concept* reveals that the main dimension to be considered in terms of user inactivity is *Information*, in view of the existing *asymmetry* between the knowledge of the users and health professionals at the PHCU. This discrepancy is noteworthy when considering the indicators *education, knowledge* and *information sources*, being important determinants to understand the steps in the HDMP flowchart and essential to prevent inactivity and increased cardiovascular risk in hypertensive (95%), diabetic (44%) and dyslipidemic patients (31%). 
